# Finite size effect on the structural and magnetic properties of MnAs/GaAs(001) patterned microstructures thin films

**DOI:** 10.1038/s41598-017-17251-y

**Published:** 2017-12-05

**Authors:** Cristian Mocuta, Daniel Bonamy, Stefan Stanescu, Souliman El Moussaoui, Antoine Barbier, François Montaigne, Francesco Maccherozzi, Ernst Bauer, Rachid Belkhou

**Affiliations:** 1 Synchrotron SOLEIL, L’Orme des Merisiers Saint Aubin, BP 48, 91192 Gif-sur-Yvette, France; 2 DRF/IRAMIS/SPEC, CEA-CNRS-Univeristy Paris Saclay, CEA-Saclay, 91191 Gif-sur-Yvette, France; 3 Institut Jean Lamour, Université de Lorraine - CNRS, BP70239, 54506 Vandoeuvre lés Nancy, France; 4 Diamond Light Source, Harwell Science and Innovation Campus, Didcot, Oxfordshire OX11 0DE United Kingdom; 5 Department of Physics, Arizona State University, Tempe, AZ 85287-1504 USA; 6 Present Address: College of Science and Technology, Nihon University, 24-1 Narashinodai 7-chome, Funabashi-shi, Chiba 274-8501 Japan

## Abstract

MnAs epitaxial thin films on GaAs(001) single crystalline substrates crystallize at room temperature (RT) in a mixture of two crystalline phases with distinct magnetic properties, organized as stripes along the MnAs [0001] direction. This particular morphology is driven by anisotropic epitaxial strain. We elucidate here the physical mechanisms at the origin of size reduction effect on the MnAs crystalline phase transition. We investigated the structural and magnetic changes in MnAs patterned microstructures (confined geometry) when the lateral dimension is reduced to values close to the periodicity and width of the stripes observed in continuous films. The effects of the microstructure’s lateral size, shape and orientation (with respect to the MnAs $\mathrm{[11}\bar{2}\mathrm{0]}$ direction) were characterized by local probe synchrotron X-ray diffraction (μ-XRD) using a focused X-ray beam, X-ray Magnetic Circular Dichroïsm - Photo Emission Electron Microscopy (XMCD-PEEM) and Low Energy Electron Microscopy (LEEM). Changes in the transition temperature and the crystalline phase distribution inside the microstructures are evidenced and quantitatively measured. The effect of finite size and strain relaxation on the magnetic domain structure is also discussed. Counter-intuitively, we demonstrate here that below a critical microstructure size, bulk MnAs structural and magnetic properties are restored. To support our observations we developed, tested and validated a model based on the size-dependence of the elastic energy and strain relaxation to explain this phase re-distribution in laterally confined geometry.

## Introduction

**Figure 1:**
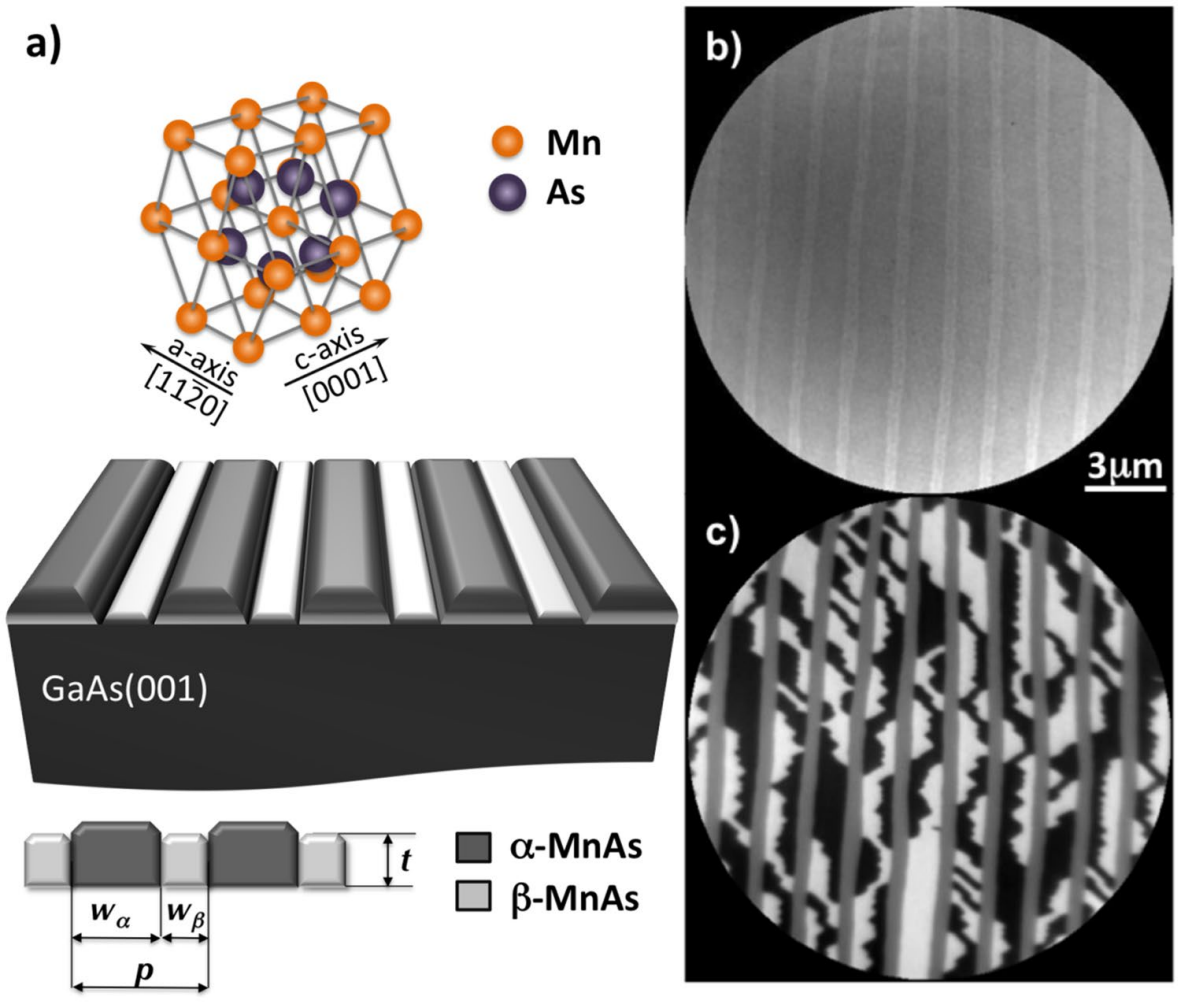
LEEM and XMCD-XPEEM images of a 300 nm thick MnAs sample. (**a**) The sketch illustrates the surface morphology and structure of the MnAs thin films at room temperature. (**b**) LEEM image taken with a primary electron energy of 11eV. The bright area corresponds to the *β*-phase. (**c**) XMCD-PEEM image at the *L*_3_ Mn edge showing the ferromagnetic MnAs domains in the *α*-phase and the paramagnetic domain in the *β*-phase.

MnAs is a promising candidate for electrical spin injection into GaAs and Si based semiconductors^1–3^. Indeed, it has a large carrier spin polarization, small coercive field and relatively high saturation magnetization and Curie temperature. Bulk MnAs crystals are known to exhibit a hexagonal (*α*-phase) ferromagnetic structure at low temperature and to experience a first order phase transition to the paramagnetic distorted orthorhombic *β*-phase above a critical temperature T_c_, approximately around 45 °C^4,5^. Epitaxial MnAs films on GaAs single crystalline substrates^6–10^, which are more appropriate for spin injection applications, show the coexistence of both the aforementioned phases (α and *β*) at RT and over a more or less extended temperature range that depends on the film characteristics (thickness and orientation) and manufacturing conditions. The equilibrium coexistence observed in this case^11,12^ was shown to result from the large mismatch between the *α*-MnAs and GaAs lattice spacing along the $\mathrm{[11}\bar{2}\mathrm{0]}$ direction of MnAs (a-axis), which is parallel to the GaAs $[\bar{1}\mathrm{10]}$ direction. This large anisotropic lattice mismatch yields epitaxial strain that the system relieves by inserting over a large temperature range around RT *β*-MnAs domains which have a smaller lattice mismatch with the substrate. The lattice parameter discontinuity between *α* and *β*-phases translates into the onset of a characteristic stripe pattern of alternating ridges and grooves along the MnAs $\mathrm{[11}\bar{2}\mathrm{0]}$ direction^5,13^ (see Fig. 1a). Several ways of imaging and characterizing these structures have been used, including Magnetic Force Microscopy (MFM)^6,14^, XMCD-PEEM and LEEM microscopies^7,15–19^.

A potential use of such a system in microelectronics and device manufacturing requires miniaturization, down to micron and sub-micron sizes. Size reduction can be beneficial because of the occurrence of genuine material properties that can be exploited in new devices or detrimental by altering or suppressing desirable bulk properties. It is thus necessary to characterize and to quantify the MnAs film properties in laterally confined geometries, namely when the lateral size approaches the stripe periodicity *p*. Generally speaking, patterned samples can have very different micro-crystalline and micromagnetic behavior than continuous thin films, and therefore one cannot simply extrapolate the results of thin films to microstructured patterns due to finite size effects^20–23^. In the specific case of the MnAs system several important questions remain open: How the crystalline phases (and consequently the magnetic domains) will organize when the associated energies become smaller than the thermal energy? What is the effect of the lateral finite size on the strain release, and consequently, on the coexistence regime of the *α*/*β* crystalline phases?

Moreover, the high uniaxial anisotropy present in this system along MnAs $\mathrm{[11}\bar{2}\mathrm{0]}$ direction requires also an investigation of a possible influence of the orientation of the confined microstructures with respect to the crystallographic directions. This issue remains challenging and has already been raised in the studies of MnAs ribbons by Tortarolo *et al*.^14^, disks by Takagaki *et al*.^22^ both using MFM and by Steren *et al*.^24^ on thin MnAs ribbons using XMCD-PEEM.

We report here on our recent investigation of the size effect on the magnetic and structural properties of microstructured MnAs patterned thin films. The MnAs system, even in bulk form, is known to evidence a strong structural and magnetic correlation. A dual approach giving simultaneously access to both physical properties on the very same objects is mandatory to get solid insights in the underlying physical mechanisms. Therefore, we have used an original combination of two local probe X-ray methods: *μ*-probe X-Ray Diffraction (*μ*-XRD) and XMCD-PEEM to investigate the finite size effect on the magneto-structural properties. *μ*-XRD allows to directly access the local lattice parameters and unambiguously identify and quantify the presence of the two crystalline phases for microstructured MnAs thin films. Direct quantitative access to the strain is also granted by this technique. XMCD-PEEM microscopy has been used to evidence the effect of the finite size effect and lateral confinement on the magnetic properties of the MnAs *α*-phase.

To corroborate our experimental observations, we propose and validate a model originally inspired by that of Kaganer *et al*.^11,12^, which consists in taking into account the size-dependence of the elastic energy stored globally in the microstructure to describe the *α*/*β* phases coexistence diagram in a 300 nm thick patterned MnAs thin film. This model is found to reproduce fairly well our observations.

## Results

### MnAs Thin film case

We begin with a brief overview of some of the results from LEEM and XMCD-PEEM studies of continuous MnAs thin films, that are relevant for the present report^7,25,26^. As stressed in the introduction, two magnetic and structural phases coexist in MnAs thin films: ferromagnetic hexagonal *α*-MnAs and orthorhombic *β*-MnAs. The coexistence is due to the anisotropic strain caused by the strong expansion of the MnAs basal plane during the phase transition from the high temperature *β*-phase to the low temperature *α*-phase, which in the bulk occurs with a small hysteresis around 45 °C. The strain is predominantly uniaxial leading to the formation of alternating stripes of *α* and *β*-phases, with the stripes direction perpendicular to the basal plane, which is perpendicular to the film surface (See Fig. 1).

The width of the *α* and *β* stripes (*w*_*α*_ and *w*_*β*_) depends both on the temperature and film thickness (*t*), while the period of the stripes (*p* = *w*_*α*_ + *w*_*β*_) increases linearly with the thickness ($p\underline{ \sim }4.8t$) and does not depend on the temperature. The strain induced by the strong lattice mismatch with the GaAs substrate is strongly temperature dependent. Thus, the MnAs film adopts a pure *β*-phase at elevated temperature (T $\gtrsim$ 100 °C), and pure *α*-phase at low temperature (T $\mathop{ < }\limits_{ \tilde {}}$ 10 °C). The phase coexistence range is directly linked to the strain relaxation and thus to the film thickness and the growth conditions. Finally and due to the large atomic lattice parameter difference between the hexagonal and the orthorhombic phase, a 1.7% corrugation with respect to the film thickness, is reported within the phase coexistence temperature range.

From the magnetic point of view, *α*-MnAs has a large negative magnetocrystalline anisotropy with three easy $\mathrm{ < 11}\bar{2}\mathrm{0 > }$ axis in the basal plane and a hard axis perpendicular to it (c-axis). As a consequence the magnetization is pointing not along the stripe direction as expected from shape anisotropy considerations but rather perpendicular to it, predominantly in-plane. This leads to a complicated, thickness-dependent magnetic domain structure in the interior of the *α*-phase^27^ which is reflected in the complexity of the magnetic images of the surface of the film (see Fig. 1c). The key point to understand the magnetic domain onset in MnAs thin films is to consider the stability of the three-dimensional magnetization distribution. Indeed, starting from a critical thickness ($\gtrsim$100 nm), the demagnetization energy induced by the lateral confinement of the *α*-phase, is reduced by the formation of 3D flux-closure domains at the cost of the exchange energy. Three magnetic configurations are generally observed: Type I (S and/or Landau states), type II (diamond state) and type III (double diamond state). The prevalence of three domain configurations depends on the film thickness and the temperature.

The XMCD-PEEM (see methods) image reported in Fig. 1c shows the micromagnetic structure of such a film. The ferromagnetic domain structure of the *α*-MnAs phase is revealed by circular dichrosm imaging^28^. In Fig. 1c, the black/white contrast results from ferromagnetic domains with opposite magnetization perpendicular to the direction of the *α* stripes and parallel to the direction of the incoming photon light; while the gray stripe contrast corresponds to the paramagnetic *β*-MnAs.

### *α-β* structural phase coexistence in MnAs microstructures

**Figure 2:**
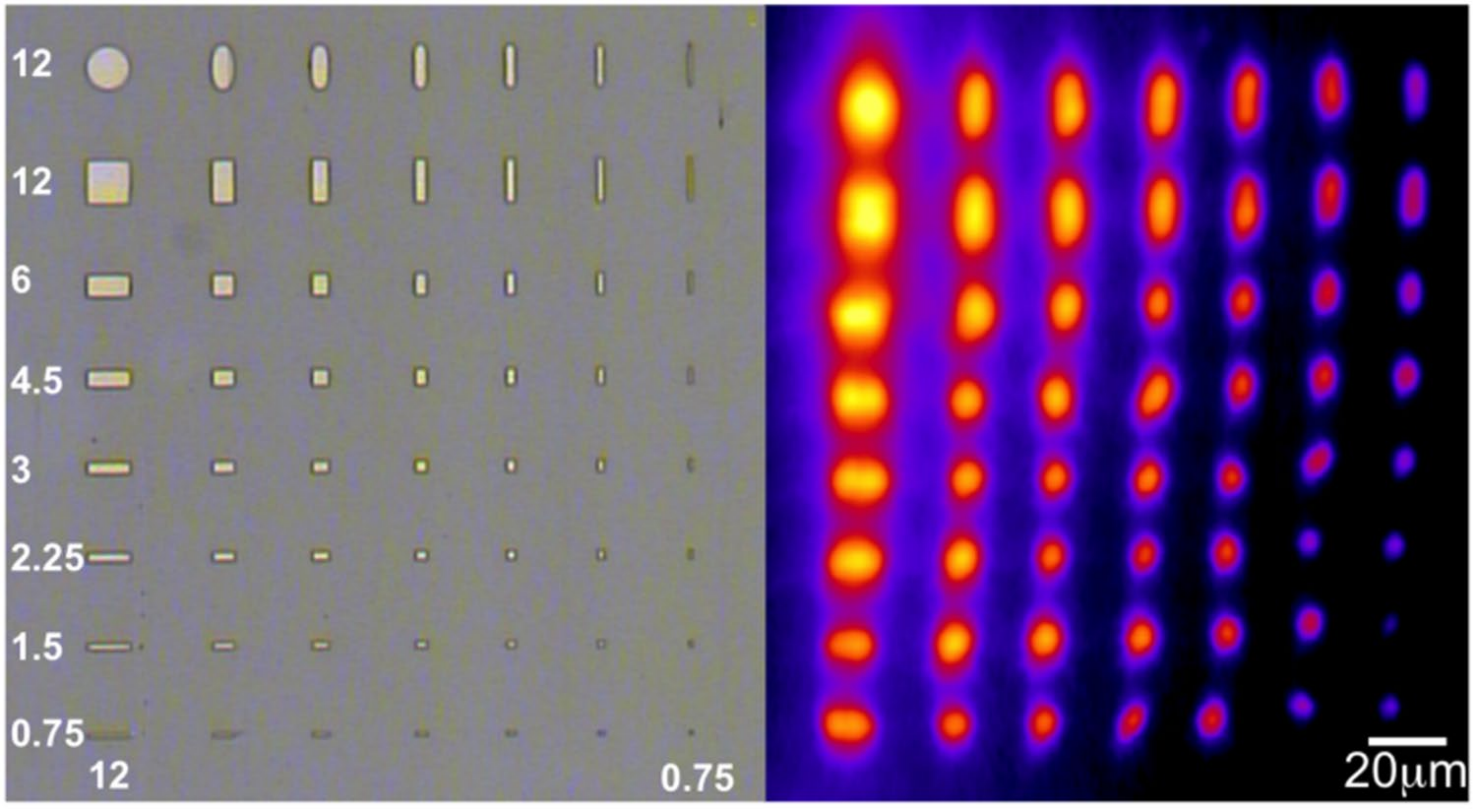
Comparison between optical microscopy images (left) and corresponding low resolution *μ*-XRD raster map (right) of rectangular MnAs patterns. The microstructure sizes in vertical and horizontal directions range from 0.75 *μm* to 12 *μm*. The *μ*-XRD raster map serves to localize the microstructures. The low resolution and distortions in it are in part due to the larger size of the X-ray spot along its direction of incidence (*θ*_*i*_ ≈ 24°), in part due to the limitations of the scanning stage (drift, wobbling,), which is not specifically designed for the large image scan.

The films used in this study were prepared by solid-source molecular beam epitaxy^26^ (MBE, see methods). MnAs films of 300 nm thicknesses were epitaxially grown on a GaAs(001) single crystalline substrate. The samples were patterned by electron beam lithography with rectangular and elliptical shaped microstructures; the 2D lateral sizes of the microstructures are ranging from 12 *μm* down to 0.75 *μm* (this last one being half of the *α*/*β* stripes period (*p*)) (Lithography, see methods). The aspect ratio and the orientation with respect to the MnAs $\mathrm{[11}\bar{2}\mathrm{0]}$ direction were also varied (Fig. 2). *L*_*a*_ refers to the size of the microstructure along the $\mathrm{[11}\bar{2}\mathrm{0]}$ direction (a-axis), while *L*_*c*_ corresponds to the size in the orthogonal direction (c-axis). This particular arrangement ensures having on the same sample rectangles and/or ellipses with the long dimension either parallel or perpendicular to the large strain direction.

**Figure 3:**
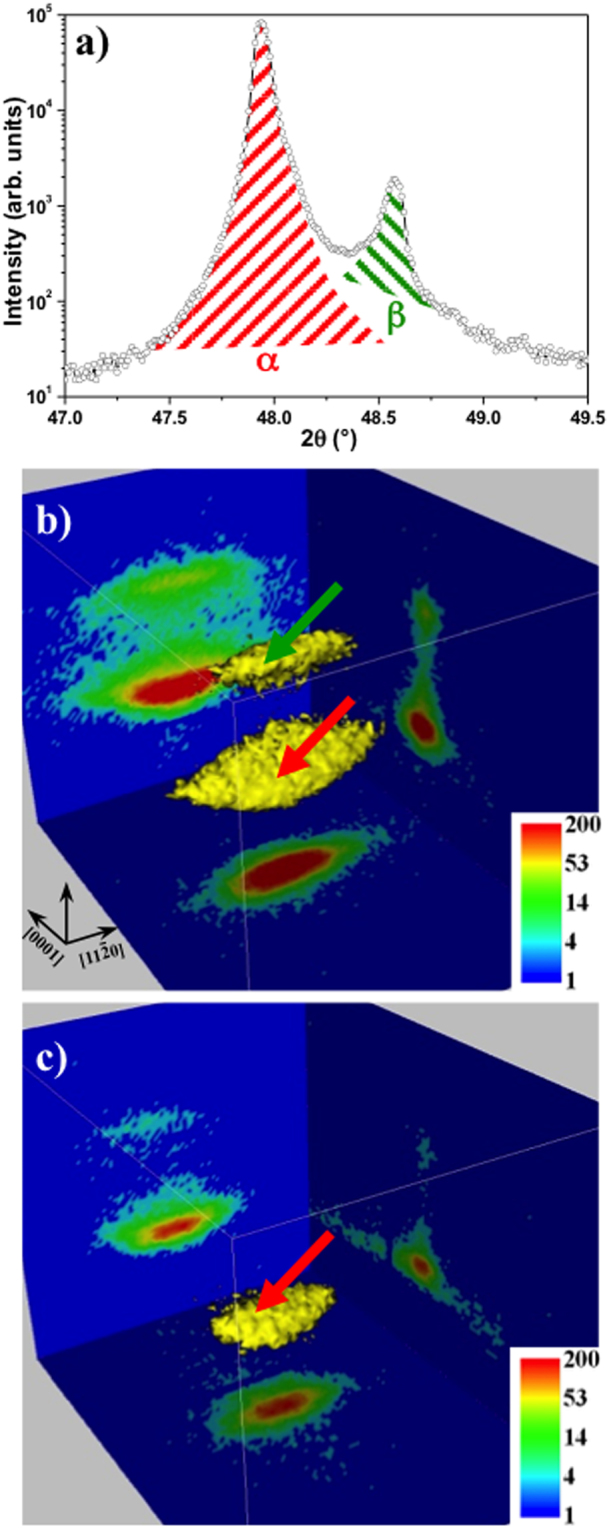
(**a**) θ − 2θ scans through the MnAs characteristic peaks. The quantity of *α* and *β* phase is estimated by calculating the integrated intensity characteristic for each phase and corrected by the corresponding structure factor. (**b**,**c**) representation of the reciprocal space around the Bragg peaks characteristic of the MnAs *α* and *β* phases (indicated by the red and green arrows respectively) for respectively 12 × 12 *μm* ^2^ and 4.5 × 4.5 *μm* ^2^ MnAs microstructures. The color scale bar corresponds to the Bragg peaks intensity.

Before any measurements, the sample was first annealed at a temperature high enough to pass through the phase transition towards the pure *β*-MnAs phase for all MnAs lithographed microstructures. This procedure resets the system and allows avoiding measuring particular “frozen-in” configurations resulting from the lithography process. The sample is then gently cooled down to RT and *μ*-XRD measurements are performed. Figure 3 shows a *θ* − 2*θ* scan across the Bragg peaks characteristic for the *α* and *β*-MnAs phases, using a hybrid pixel detector (X-ray energy E = 9.5 keV). The very sharp and intense GaAs(001) substrate Bragg peak (not shown) was found at 2*θ* ≈ 54.94°, as expected from the bulk GaAs lattice parameter value. The quantity of the *α* and *β* phases can be estimated by calculating the integrated areas of the peaks (hatched regions in red and green for *α* and *β* phase, respectively), and correcting the result with the appropriate structure factor. This simplified approach allows a rapid estimation, but does not take into account a possible broadening of the peaks in the *θ* direction. For the refined data acquisition, the approach is extended in the following way: each MnAs microstructure is centered in the X-ray beam by laterally scanning its position in the x and y directions (Fig. 3a) using the XRD signal as contrast. Then the incident angle (*θ*_*i*_ = *θ*_*B*_) is scanned around the corresponding Bragg value, recording the full image of the area detector. The data-set obtained corresponds thus, for each MnAs illuminated microstructure, to a volume in the reciprocal space close to that Bragg peaks.

Figure 3b,c show representations of such volumes around the Bragg peaks of *α* and *β*-MnAs for two different microstructure sizes: 12 × 12 and 4.5 × 4.5 *μm* ^2^, respectively, measured at RT. The same absolute color scale is used for the scattered intensity. The representation consists of an iso-intensity surface and three planar cuts through the peaks, along high symmetry planes. The expected positions of the Bragg peaks of *α* and *β* phases are pointed out by red and green arrows respectively. One can note the much lower absolute maximum intensity in panel c), a natural consequence of a smaller microstructure (volume) illuminated by the X-ray beam. Also note the change of the fraction of *α*-MnAs phase (from 89% to 98%) when the lateral size of the microstructure is reduced. A faint but negligible trace of the *β*-MnAs Bragg peak can still be detected in Fig. 3c for the small microstructure, see the vertical plane cuts.

**Figure 4:**
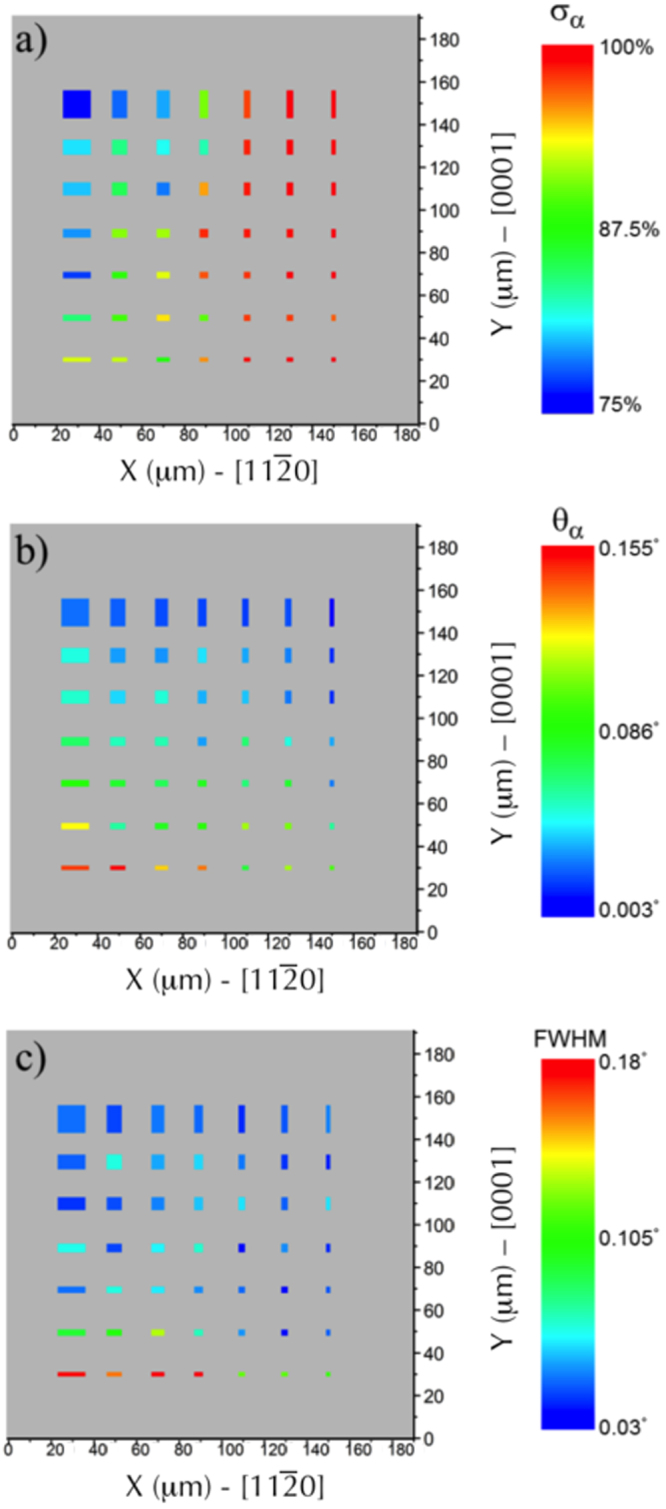
Evolution of the structural parameters of the MnAs microstructures at RT, as function of the lateral size in the two orthogonal directions (*L*_*a*_ and *L*_*c*_). (**a**) The *α* fraction varies from 75% for the largest microstructure (12 × 12 *μm* ^2^) to 100% for the smallest one (0.75 × 0.75 *μm* ^2^). The peak position shift (related to the lattice parameter normal to the surface) and its width are also extracted for each lithographed microstructure and are shown in panels (b) and (c) (see text for details). The *α*/*β* stripes are oriented perpendicular to the impinging X-ray beam.

This approach allows not only to estimate the ratio of *α*/*β* phases, but to extract as well the 2*θ* positions of the characteristic Bragg peaks and their corresponding widths, for each MnAs microstructure. The Bragg peak position is related to the lattice spacing (in the direction perpendicular to the surface in this particular case, *i*.*e*. the MnAs $\mathrm{[1}\bar{1}\mathrm{00]}$ direction), so also to the strain. The width of the peak can be related to the presence of local crystalline defects in the probed volume. These values are reported, at RT, in Fig. 4a,c as color graphics: each bar represents the shape and size of the lithographed microstructure, while the color (from blue to red) is related to the amplitude of the reported quantity: *α*-phase fraction (*σ*_*α*_), the relative position (*θ*_*α*_) and the full width at half maximum (FWHM) of the *α*-phase Bragg peak.

The results reported and discussed here concern several set of measurements and samples (patterns), including rectangular and elliptical shapes. The elliptic microstructures were intended to investigate the effect of corner-induced magnetic stray fields on the magnetic domain structure. From our XRD structural measurements we didn’t observed any effect related to the shape of the microstructures. Therefore, we will discuss here only the rectangular shaped patterns.

### Effect of size reduction on the *α-β* phases repartition at RT

The reference quantity *σ* ^∞^ of the *α*-phase fraction for a continuous MnAs film (non-lithographed part of the sample) was measured by XRD to be around 72.7% at RT, which is close to the value found for the 12 × 12 *μm* ^2^ microstructure. Thus, the former may be quite representative of the infinite microstructures.

When exploring microstructures with the long dimension *L*_*c*_ perpendicular to the *α*/*β* stripes (*i*.*e*. this dimension is parallel to the X-ray direction and corresponds to the low strain direction), a significant quantity of *β*-phase is still present. We can phenomenologically understand this finding by the fact that a ‘long dimension’ allows accommodating several stripes (*i*.*e*. alternating *α*/*β* phases) and thus include the *β*-phase.

When the lateral dimension of the MnAs microstructure is reduced in the direction parallel to the *α*/*β* stripes (*L*_*a*_), the *α*/*β* phases ratio increases. The pure *α*-phase is found for microstructures having the lateral size smaller than the stripe period *p* (${L}_{a}^{c}\,\simeq \,4.8t$). This value is in agreement with the conclusions of Tortarolo *et al*.^14^ in the case of MnAs ribbons (${L}_{a}^{c}\, > \,$3.2t).

### Effect of size reduction on the *α-β* structure at RT

The shift in the 2*θ* position is calculated with respect to the position found in the continuous MnAs film case, for which we can confidently assume that the *α*/*β* domains are unaffected by any lateral confinement-like effect (infinite film case). A non-lithographed part of the sample of 200 × 200 *μm* ^2^ was used for this purpose.

The largest shift of the position of the peak is obtained for the rectangular shapes (smaller width, large *L*_*c*_) oriented along the X-ray beam (*i*.*e*. which are crossed by many stripes). Combined with the result reported for the *α*/*β* phases ratio, the shift can be phenomenologically explained as follows: these microstructures can accommodate many *α*/*β* stripes, thus their crystalline structure will be affected. The ‘average’ lattice parameter of the *α*-phase tends to adapt to the *β*-phase one, yielding thus a significant shift in the 2*θ* position of the Bragg peak. A similar situation is happening for the *β*-phase characteristic peak (not shown here). These microstructures exhibit the presence of more crystalline defects, causing a broadening of the Bragg peak (mosaicity like). A similar effect can be seen on the peak characteristic for the *β*-phase. Due to the low fraction of the *β*-phase, and consequently peaks with lower intensities, the corresponding data are noisier (see e.g. Fig. 3a).

In the case of the rectangles oriented with the long axis along the stripes direction (small *L*_*a*_), they will simply accommodate a single phase, with a clear preference for the *α*-phase (at RT), as shown by the graph in Fig. 4a.

Although we have focused in this paper on size effects when varying the objects lateral dimension along the $\mathrm{[11}\bar{2}\mathrm{0]}$ direction (*L*_*a*_), it is interesting to note that differences are also detected when the object size is varying along the [0001] direction (*L*_*c*_). For both, the position of the *α* phase Bragg peak (*θ*_*α*_) and its FWHM (Fig. 4b,c), there is a significant increase of their values when *L*_*c*_ is decreasing. These results suggest that the *α* phase exhibits more local defects when *L*_*c*_ is reduced. We propose the following possible explanation: It has been already shown, for a sample made of epitaxial layers, that significant changes in crystalline lattice parameter and lattice plane orientation can appear in the vicinity of the edges of the lithographed object^29–32^. For the objects mentioned here, when decreasing *L*_*c*_ (and large *L*_*a*_), the weight of the border area (*i*.*e*. significant perturbation of the lattice) becomes increasingly important in the sample area. This translates into a shift and increase of *θ*_*α*_ and FWHM values respectively, reflected by the variations reported in Fig. 4b,c. Note that when *L*_*a*_ is getting smaller (comparable to the stripes period), the above mentioned effect might have a lower amplitude: since only one or two stripes can be accommodated inside the object, this may somehow lock the crystalline phase and leaves less space for introducing relaxation and/or defects.

### Effect of size reduction on the *α−β*-phase transition

**Figure 5:**
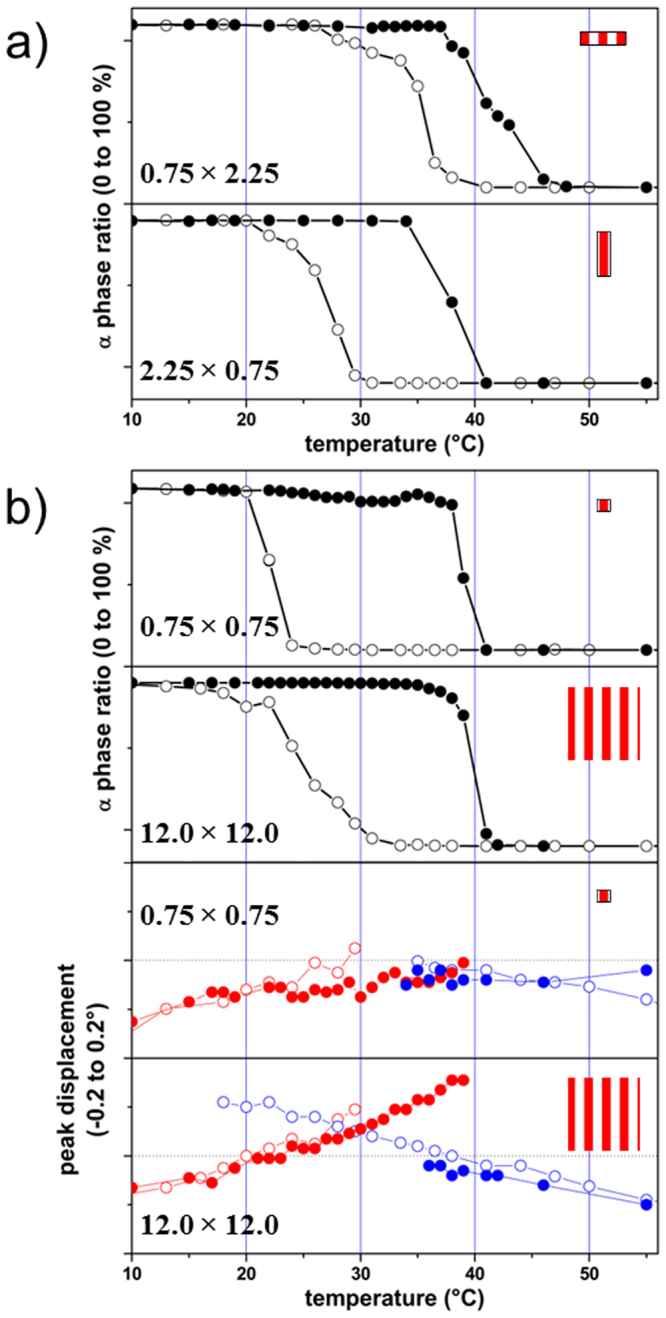
Fraction of the *α*-phase as a function of sample temperature for two orthogonal MnAs microstructures (**a**) 0.75 × 2.25 *μm* ^2^ and 2.25 × 0.75 *μm* ^2^ and two isotropic ones (**b**) -top: 0.75 × 0.75 *μm* ^2^ and 12 × 12 *μm* ^2^. The first dimension reported for each microstructure is the one along the beam direction and perpendicular to the stripes (*L*_*a*_: see also the inset, not at scale). The heating (filled symbols) and cooling (open symbols) curves are shown. (**b**) -bottom: Shift of the characteristic Bragg peak position of the isotropic microstructures, corresponding to the *α* (red) and *β* (blue) phases respectively, for heating (filled symbols) and cooling down (open symbols).

We performed local probe *μ*-XRD experiments at various temperatures across the *α*/*β* phase transition, in order to access the possible dependence of the critical transition temperature *T*_*c*_ and the phase coexistence temperature range, on the size and shape of the MnAs microstructures. All the microstructures were measured at about 40 temperatures (in the range of 8 to 55 °C) in order to cover the full phase transition region. Quantities similar to the ones reported at RT in Fig. 4a,c are extracted, and their change with temperature is shown in Fig. 5.

These results show first the presence of a thermal hysteresis loop: the phase transition does not happen at the same temperature when heating or cooling the sample. A significant change of *T*_*c*_ with microstructure size is also noticed. However it is difficult to extract a clear quantitative dependence on the lateral size. This is most likely due to the initial growth conditions and to the lithography process.

As a matter of fact, because of the large anisotropic lattice mismatch between GaAs and MnAs, a large stress field forming array of misfit dislocation^33^ is created at the interface during the initial stage of the growth and propagates through the whole thickness of MnAs sample. The stress filed is not released with further thermal processing. In the case of the continuous film and because of this stress field, the *α*/*β* stripes always appear at the same position, whatever the thermal history *i*.*e*. cooling down from the pure *β*-phase to the pure *α*-phase and *vice versa*. However, the microstructures have been randomly lithographed over the sample surface with respect to the strain field in the film, cracks and defects. Therefore and depending on the nucleation position of the *α*/*β* boundary with respect to the position of the microstructure and to defects, the thermal hysteresis can evidence different behavior.

Nevertheless, and without outrunning the above mentioned influence of particular sample history and preparation, an important effect is highlighted for rectangular microstructures of the same size but oriented parallel and perpendicular to the *α*/*β* stripes (low and large strain direction, respectively). For the microstructures oriented with the long dimension perpendicular to the stripe direction, the *β*-phase appears earlier during cooling down (e.g. the 0.75 × 2.25 *μm* ^2^ curve) than when the long dimension is parallel to the stripe direction (e.g. the 2.25 × 0.75 *μm* ^2^ curve). We could understand this by noting that the microstructure with the long dimension along the stripes can contain less *β*-phase at RT and, consequently, the *β*-phase appears at lower temperature. As mentioned above, the presence of crystalline defects especially close to the edges of the objects (tilts of crystalline planes and relaxation^29–31^) is likely. So we can assume they constitute nucleation/pinning centers for the alternating *α*/*β* stripes, provided that the lateral size of the object along the $\mathrm{[11}\bar{2}\mathrm{0]}$ direction is larger than a critical one (see below). It is known that epitaxial clamping effects can substantially change transition temperatures^34^ for thin films. If, in addition, only one phase accommodated inside the object, the transition sharpness may also be affected, as it can be seen for example in Fig. 5a (bottom panel) and 5-b (top panel).

The most important result from the temperature study is the reduction of the temperature range of the *α*/*β* phases coexistence. While in the continuous film, this temperature range extends over several ten of degrees, it is reduced to only a few degrees for microstructures having a lateral size below the critical length ${L}_{a}^{c}\le 1.5\,\mu m$ along the large strain direction. This result is very important because it demonstrates, as it will be further confirmed by the theoretical modeling, that size reduction along the high strain direction, allows to relax the large anisotropic strain which is the main cause of the phase coexistence in the continuous film.

The shift of the position of the Bragg peak (Fig. 5b, bottom panels) is representative for the strain variation in the MnAs films; the thermal expansion is much smaller in this temperature range, and is neglected. The curves corresponding to *α* and *β* phases (Bragg peaks) cross at zero shift (with respect to the values found for the reference MnAs continuous film), which is always almost centered with respect to the hysteresis loops discussed above.

These temperature and size dependent results confirm that the phase transition in laterally confined MnAs structures is governed by uniaxial anisotropic strain.

### Magnetic domain structure of MnAs microstructures

The *μ*-XRD results have been further confirmed by RT LEEM and XMCD-PEEM measurements of rectangle, disk and elliptic shapes, performed on the same sample. As observed with *μ*-XRD and as it will be discussed hereafter, the stabilization of the *α*/*β* stripes is different in the laterally confined MnAs microstructures as compared to the MnAs continuous films of the same thickness.

First of all, it is worth to stress that the two methods allow accessing different sample probing depths. XPEEM is essentially surface sensitive and thus allows to investigate the domain structure of the first few nanometers of the sample. *μ*-XRD has a larger penetration depth (a few 100 nm) and thus provides more bulk information. Therefore, the combination of these two local probe methods allows investigating the strain relaxation effect and its close relationship to the finite size effect both in the bulk and at the surface of the MnAs microstructures. At the surface, strain relaxation can occur due to the broken bonds and thus modify the surface elastic energy of the film. Consequently, the surface *vs* bulk *α*/*β* phases repartition can be very different. In addition, the XMCD-PEEM measurements allow accessing the magnetic configuration of the *α*-phase and thus correlating the structural and magnetic properties.

**Figure 6:**
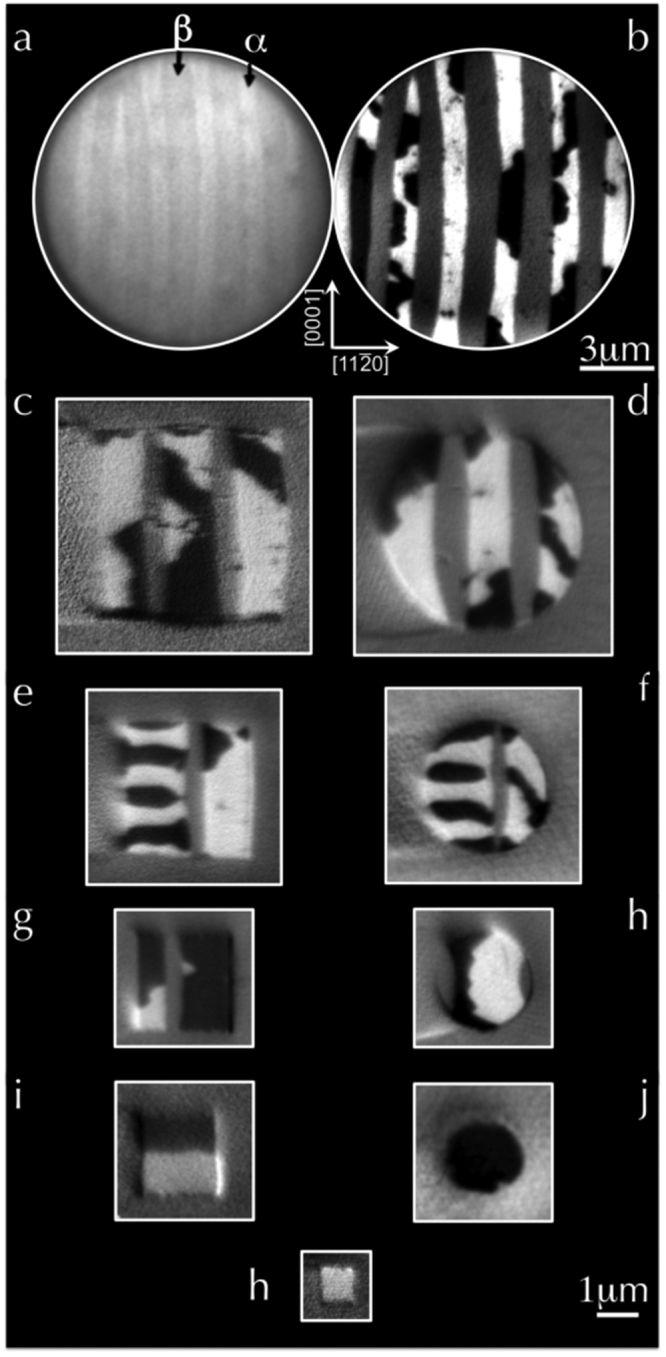
Selected LEEM and XMCD-PEEM images of isotropic MnAs microstructures (disks and squares): (**a**) 12 *μm* disk LEEM image evidencing the recovery of good surface crystalline structure after the *in-situ* preparation and the lithography process. (**b**–**k**) Selected XMCD-XPEEM images for various microstructure sizes and shapes: (**b**) 12 *μm*. (**c**) and (**d**) 4.5 *μm*. (**e**) and (**f**) 3 *μm*. (**g**) and (**h**) 2.25 *μm*. (**i**) and (**j**) 1.5 *μm*. (**h**) 750 nm. The images show the evolution of the magnetic domain structure and the *α*/*β* phases repartition as function of microstructure size. The black/white regions correspond to *α*-phase ferromagnetic domains with opposite magnetic moment. The gray regions correspond to the paramagnetic *β*-phase.

For the XMCD-PEEM measurements, the samples have been prepared at high temperature before cooling down to RT (see LEEM-PEEM microscopy, methods). In Fig. 6 we present XMCD-PEEM images of selected MnAs isotropic microstructures having different sizes and shapes. First, one may note that the easy magnetic direction remains along the $\mathrm{[11}\bar{2}\mathrm{0]}$ direction for all the investigated microstructure sizes and shapes. This finding confirms that the magnetocrystalline anisotropy is strong enough to overcome the magnetic shape anisotropy when reducing the lateral size of the microstructure as observed in the thin film case.

It is also worthwhile to notice, that as far as the *α*/*β* phases repartition is concerned, we did not observe any effect related to the shape of the isotropic microstructure (*i*.*e*. disk or square). Nevertheless, looking more closely at the shape of the stripe boundaries, we may note that in disk-shaped microstructures, the stripe boundaries are strongly influenced by the shape specially when the edge curvature is large. The stripe boundaries are no longer aligned parallel to the $\mathrm{[11}\bar{2}\mathrm{0]}$ direction, but tend to rotate to match perpendicularly the edge of the microstructures (see Fig. 6h). This effect can be easily understood if we consider edge effects. In general and as it will be discussed in the modeling section, the stress vector will always tend to cross perpendicularly a free surface.

As has been deduced from the *μ*-XRD measurements, the XMCD-PEEM images confirm that below a critical lateral size ${L}_{a}^{c}=1.5\,\mu m$ along the $\mathrm{[11}\bar{2}\mathrm{0]}$ direction, the microstructures adopt exclusively the *α*-MnAs phase at RT. Importantly, this corresponds to the bulk behavior that can here unexpectedly be restored by lateral size reduction. Finally, comparing the *μ*-XRD and XMCD-PEEM results allows us to conclude that there are no surface *vs* bulk effects and that the microstructures are homogeneous through their whole thickness.

From the magnetic point of view, a similar behavior with respect to the size effect has been evidenced. Below 2.25 *μm* the magnetic domains start to differ from those observed in the continuous thin film and do not evidence the characteristic zig-zag domains structure^25^. The microstructures develop a magnetic domain structure similar to the one observed in the low temperature pure *α*-phase^26^. The 1.5 *μm* microstructure develops single and head-on domains, while the smallest microstructures (0.75 *μm*) show almost exclusively single magnetic domains.

**Figure 7:**
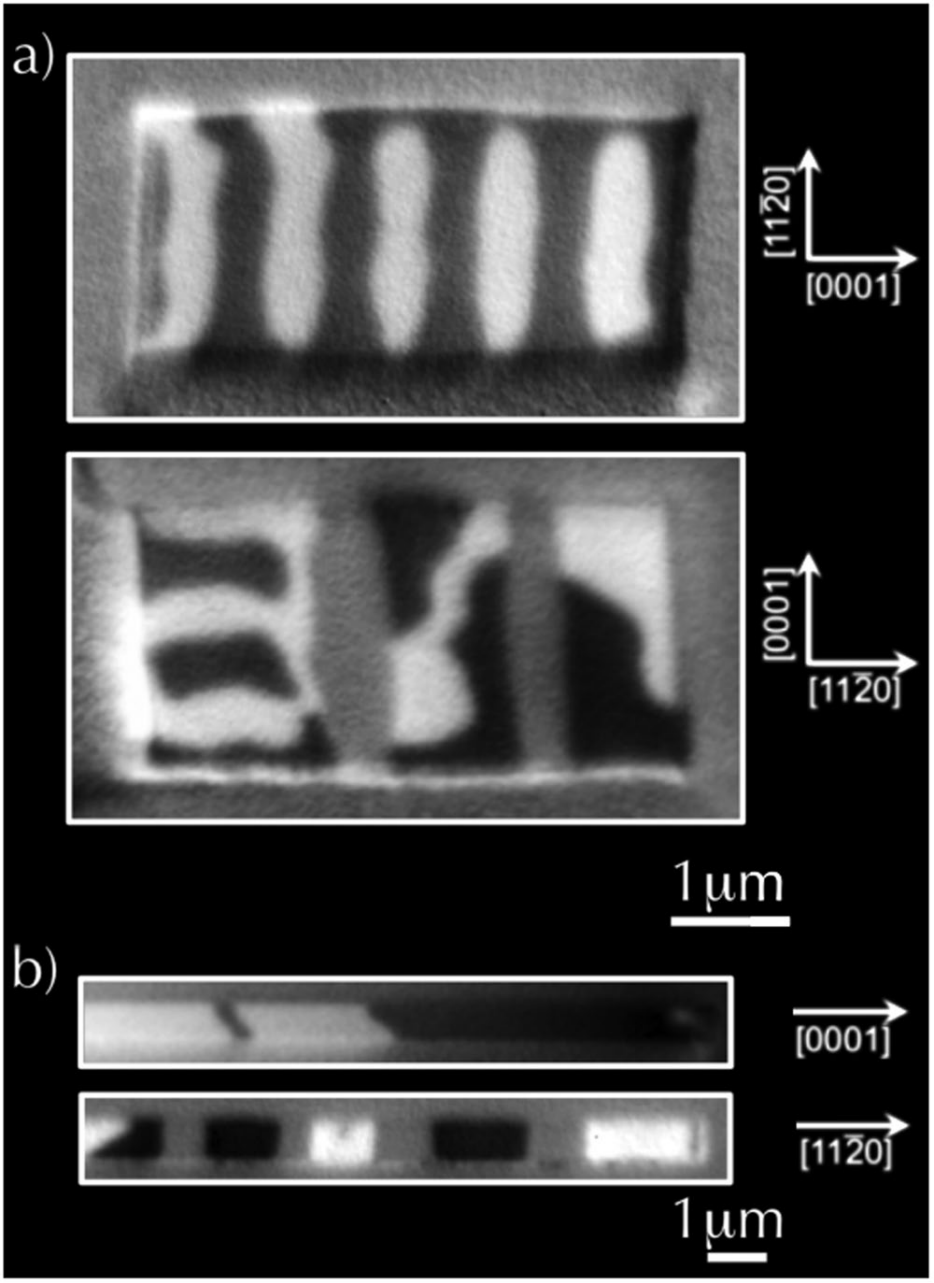
XMCD-XPEEM images for orthogonal MnAs rectangular microstructures: (**a**) 4.5 × 2.25 *μm* ^2^ (top) and 2.25 × 4.5 *μm* ^2^ (bottom). (**b**) 12 × 0.75 *μm* ^2^ (top) and 0.75 × 12 *μm* ^2^ (bottom). On the top (bottom) images, the long axis is aligned perpendicular (parallel) to the $\mathrm{[11}\bar{2}\mathrm{0]}$ direction.

Figure 7 shows the domain configuration of two microstructures, 2.25 × 4.5 *μm* ^2^ and 0.75 × 12 *μm* ^2^, with two different orientations with respect to the large strain direction. As expected, the rectangular structures with the long axis oriented along the [0001] direction, hereafter denoted rectangle-A, are predominately *α*-phase, while for the orthogonal microstructures (rectangle-B), the two *α*/*β* phases coexist in the form of alternating stripes. This result is in remarkable agreement with *μ*-XRD measurements.

Looking more closely at the onset of the magnetic domain structure, one may see that the rectangle-A develops elliptically shaped domain structures. Such domain structures have been already observed in continuous films and correspond to a mixture of two magnetic domains: I (S state or Landau state) and III (double diamond state)^25,27^. Their occurrence in continuous thin film is very limited while their prevalence in microstructures is quite large.

**Figure 8:**
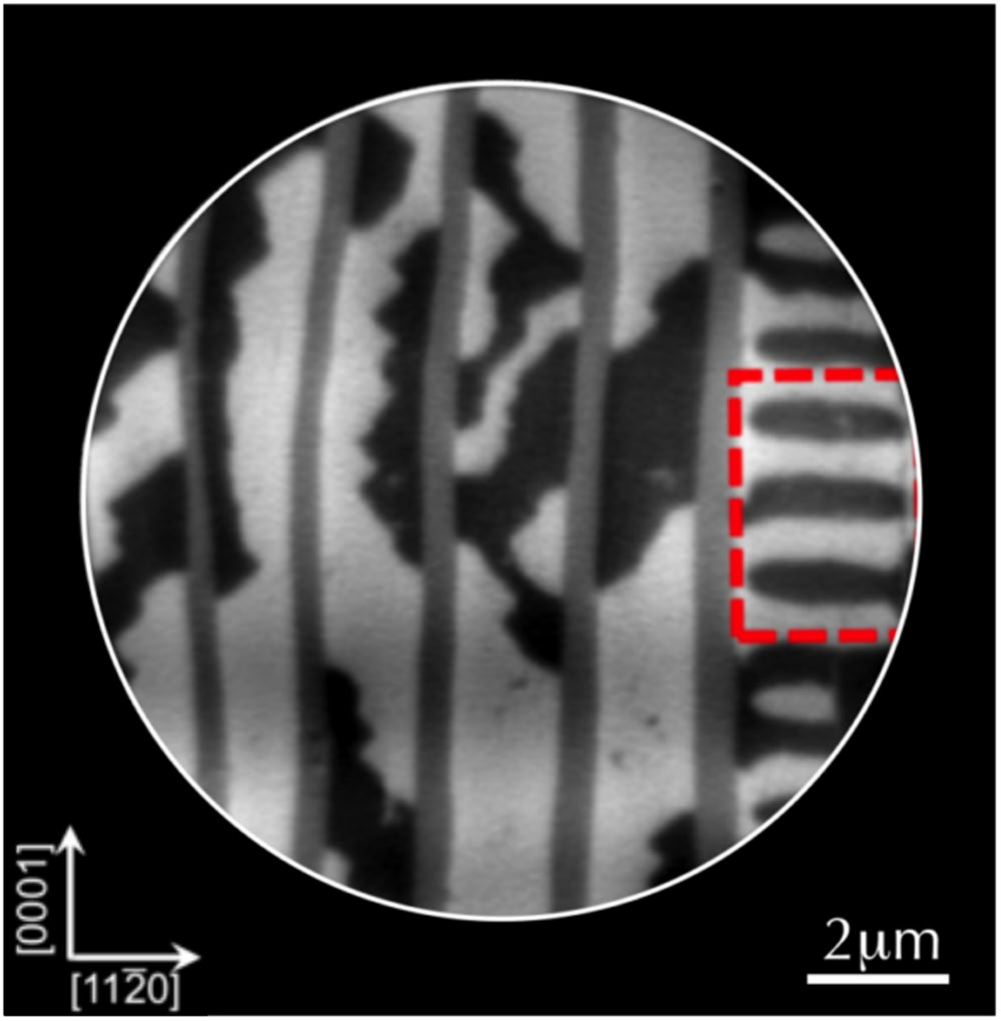
Evidence of a type I-III type magnetic domain (red dashed rectangle) in a large 12 × 12 *μm* ^2^ rectangular microstructure which often appears at the edge of the microstructure. The field of view is 10 *μm* and only a section of the microstructure is shown.

The fact that this domain state has been observed also in very large microstructures (Fig. 8) suggests that their predominance is probably not correlated with size reduction. From the statistics of the domain structure over several microstructures, the I-III domains are always observed when the ferromagnetic *α*-phase is located at the edge of the microstructures. It is therefore most likely that their stability is connected to edge effects and to the minimization of the magnetic charges at the microstructure edges via the formation of a three-dimensional flux-closure pattern at the cost of exchange energy.

Unfortunately, XMCD-PEEM allows to investigate essentially only the surface contribution of the magnetic domains and there is a real lack of methods capable to characterize the complex 3D magnetic structure of such magnetic domains. Complex 3D micromagnetic simulations are obviously needed to fully understand the origin and the stability of this particular magnetic domain structure. A detailed analysis of the micromagnetic domain structure in MnAs microstructures will be the subject of a specific publication.

### Model and theoretical interpretation

As mentioned above, the large mismatch between the hexagonal *α*-MnAs phase and the substrate GaAs lattice spacing along the $\mathrm{[11}\bar{2}\mathrm{0]}$ direction yields epitaxial strain that the system relaxes by forming *β*-MnAs domains, which exhibit smaller mismatch with GaAs. The fraction *σ* of the *α*-phase is then selected so that it minimizes the total system free energy F, which can be written as:

1 $$F={L}_{a}{L}_{c}t(\sigma {f}_{\alpha }+\mathrm{(1}-\sigma ){f}_{\beta })+{E}_{elastic}$$

where *L*_*a*_, *L*_*c*_, and *t* refer to the layer dimensions along $\mathrm{[11}\bar{2}\mathrm{0]}$, [0001], and $\mathrm{[1}\bar{1}\mathrm{00]}$ directions, respectively. *f*_*α*_ and *f*_*β*_ refer to the free energy of bulk *α* and *β* phases, respectively. *E*_*elastic*_ denotes the elastic energy stored within the MnAs layer. In the model developed here we disregard any plastic deformation and non-linear elastic phenomena. This is justified in the present system since the critical thickness for cracking is larger than the thickness of our MnAs layers^35^ (500 nm vs. 300 nm. respectively). The difference in bulk free energies *f*_*α*_ − *f*_*β*_ vanishes at the bulk transition temperature *T*_*c*_ and is expected to be a linear function of the temperature *T* close to the transition: *f*_*α*_ − *f*_*β*_ = −Q(*T* − *T*_*c*_)/*T*_*c*_, where Q is the latent heat.

**Figure 9:**
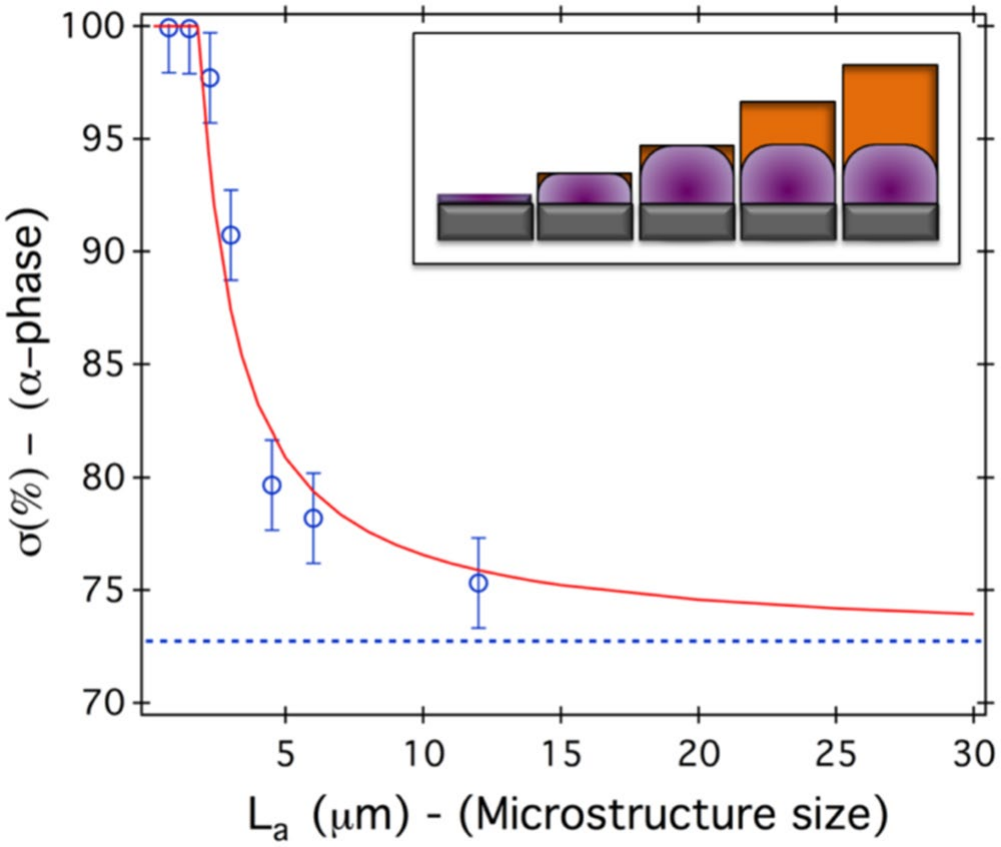
Variation of the fraction (*σ*) of the *α*-phase as a function of pillar width *L*_*a*_ as observed experimentally (open circles) and as predicted by Eq. 6 completed with the dimensionless function *f*(*u*), for a given temperature (*T*_0_ = RT). The dashed line corresponds to the experimental data from the continuous MnAs film: *σ* ^∞^ = 72.7%. This value has been used in Eq. 6 to obtain the red plain curve (no fitting parameter). Inset: Schematic drawing of the elastically strained region (purple) in MnAs layers of constant width and increasing height (orange).

Evaluation of *E*_*elastic*_ is more complex and a priori calls for a full three dimensional stress analysis. However, since the transition from *α*-MnAs to *β*-MnAs leads to a shrinking of the prism hexagon (i.e. a change in the mismatch along a-axis) without modification of the prism height (i.e. without change in the mismatch along c-axis), we choose to reduce the problem using a plane strain approximation to the simpler two dimensional problem for the stress analysis, as sketched in Fig. 9 (inset). This permits a simple semi-analytical derivation of the variations of *σ* with *L*_*a*_ and its subsequent comparison with the experimental measurements, provided that *L*_*c*_ remains large with respect to both *L*_*a*_ and *t*. The limits raised by this approximation and the effect of *L*_*c*_ will be discussed at the end of the section.

Let us first consider a MnAs film with infinite lateral dimensions. This situation is the one originally investigated by Kaganer *et al*.^11^. Then, the elastic strains are homogeneous within the film thickness and the contribution arising from the discontinuity of the lattice parameters at the *α*/*β* MnAs interfaces. Calling *ε*_*α*_ and *ε*_*β*_ the epitaxial strains at the *α*-MnAs/GaAs and *β*-MnAs/GaAs interfaces, respectively, the elastic energy densities in the *α* and *β* phases write: $Y{\varepsilon }_{\alpha }^{2}$ and *Y*(*ε*_*β*_ − *η*)^2^, respectively, where *Y* is the relevant elastic modulus (given, in first approximation, by the MnAs Young modulus). *η* is the relative change of lattice spacing along a-axis between *α* and *β* phases.

The elastic energy in Eq. 1 writes: ${E}_{elastic}={L}_{a}{L}_{c}t(\sigma Y{\varepsilon }_{\alpha }^{2}+\mathrm{(1}-\sigma )Y{({\varepsilon }_{\beta }-\eta )}^{2})$.

Then, the strains *ε*_*α*_ and *ε*_*β*_ are selected in order to minimize *E*_*elastic*_ under the constraint that the total length of the MnAs film is imposed by the GaAs substrate: *σε*_*α*_ + (1 − *σ*)*ε*_*β*_ = *ε*_0_, where *ε*_0_ is a constant set by the substrate length. This minimization process yields *ε*_*α*_ = −(1 − *σ*) + *η* + *ε*_0_, hence ${E}_{elastic}={L}_{a}{L}_{c}Y({\mathrm{(1}-\sigma )}^{2}{\eta }^{2}-{\varepsilon }_{0}^{2})$ and finally Eq. 1 writes:

2 $$\frac{{F}^{\infty }}{{L}_{a}{L}_{c}t}=(\sigma {f}_{\alpha }+\mathrm{(1}-\sigma ){f}_{\beta })+Y\mathrm{((1}-\sigma {)}^{2}{\eta }^{2}-{\varepsilon }_{0}^{2})$$

where the index ∞ has been added to F to recall a lateral dimension *L*_*a*_ infinitely large with respect to the pillar height *t*. The equilibrium density *σ* ^∞^ is the one that minimizes *F* ^∞^:

3 $$\begin{array}{lllll}{\sigma }^{\infty }(T) & = & 1 & {\rm{for}} & T < {T}_{c}^{\ast }-{\rm{\Delta }}{T}^{\infty }\\ {\sigma }^{\infty }(T) & = & \frac{Q}{2Y{\eta }^{2}}\frac{{T}_{c}^{\ast }-T}{{T}_{c}^{\ast }} & {\rm{for}} & {T}_{c}^{\ast }-{\rm{\Delta }}{T}^{\infty }\le T\le {T}_{c}^{\ast }\,{\rm{with}}\,{\rm{\Delta }}{T}^{\infty }=\frac{2Y{\eta }^{2}}{Q}{T}_{c}^{\ast }\\ {\sigma }^{\infty }(T) & = & 0 & {\rm{for}} & {T}_{c}^{\ast } < T\end{array}$$

The effect of *ε*_0_ is to shift the temperature ${T}_{c}^{\ast }$ above which the *α*-phase is no longer observed with respect to the bulk phase transition, *T*_*c*_. This simple model combined with the proper values of the material constants Y, Q, *η*, ${T}_{c}^{\ast }$ was shown^11^ to reproduce quantitatively the evolution of *σ* ^∞^ with temperature in the case of continuous MnAs thin films.

Let us now consider the effect of a finite lateral size *L*_*a*_ still keeping at this point the assumption of an arbitrary large *L*_*c*_. In this case, the elastic strains induced by the lattice mismatch between film and substrate are not homogeneous within the thickness anymore, but decrease as the distance from the film/substrate interface increases, with a characteristic decay length of the order of *L*_*a*_ (Fig. 9, inset). Two limiting cases are expected:

- In the limit of pillars of height $t\ll {L}_{a}$, the elastic energy is proportional to a volume $\simeq {L}_{a}{L}_{c}t$ of the epitaxial layer and its expression is that obtained by Kaganer *et al*.^11^ in the limit of infinite lateral dimensions.
- In the limit $t\gg {L}_{a}$, the elastically strained zone is confined in a layer of thickness $\simeq {L}_{a}$ above the interface. Hence, the elastic energy is proportional to a volume $\simeq {L}_{a}^{2}{L}_{c}$ and its relative importance with respect to bulk (volume) free energy vanishes as *L*_*a*_/*t*. One then expects to observe the same transition behavior as that observed in bulk MnAs, namely *α*-phase below *T*_*c*_ (in particular at ambient temperature *T*_0_ = *RT* < *T*_*c*_), and *β*-phase above.

In Eq. 1, the term of elastic energy should be modified to:

4 $$\begin{array}{lll}{E}_{elastic}={L}_{a}^{2}{L}_{c}Y\varepsilon \,{(y=\mathrm{0)}}^{2}f(u=\frac{t}{{L}_{a}}) & {\rm{with}} & f(u)\approx u\,{\rm{if}}\,u\ll 1\\  &  & f(u)\approx u\,{\rm{if}}\,u\gg 1\end{array}$$

where *y* is the direction perpendicular to the surface plane (*y* = 0 describes the MnAs/GaAs interface), *ε*(*y* = 0) denotes the amount of elastic strain within MnAs at the interface, and *f*(*u*) is a dimensionless function that only (and slightly) depends on the material Poisson ratio, which is well approximated by *f*(*u*) ≈ *u*_*c*_(1 − *exp*(−*u*/*u*_*c*_)) with *u*_*c*_ ≈ 0.17 (see annex for its determination). The elastic term in Eq. 2 should be modified accordingly:

5 $$\frac{F}{{L}_{a}{L}_{c}t}=(\sigma {f}_{\alpha }+\mathrm{(1}-\sigma ){f}_{\beta })+\frac{{L}_{a}}{t}f(\frac{t}{{L}_{a}})Y\mathrm{((1}-\sigma {)}^{2}){\eta }^{2}-{\varepsilon }_{0}^{2}$$

and the fraction of the *α*-phase now writes:

6 $$\sigma (T,\frac{t}{{L}_{a}})=\frac{t}{{L}_{a}}\frac{1}{f(t/{L}_{a})}{\sigma }^{\infty }(T)\,{\rm{for}}\,{T}_{c}^{\ast }-{\rm{\Delta }}T\le T\le {T}_{c}^{\ast }\,{\rm{with}}\,{\rm{\Delta }}T=\frac{{L}_{a}}{t}f(t/{L}_{a}){\rm{\Delta }}{T}^{\infty }$$

As shown in Fig. 9, this expression, completed with the proper dimensionless function *f*(*u*) (see methods), reproduce fairly well the experimental data, without any fitting parameters.

The above derivations considered that *L*_*c*_ was large with respect to both *t* and *L*_*a*_. Taking quantitatively its effect into account would require a full-three dimensional analysis of the elastic problem beyond the scope of this paper. A qualitative picture can however be proposed by noting that, in a layer of size *L*_*c*_ × *L*_*a*_ × *t*, the elastic strains induced by the lattice mismatch decrease with the distance from the film/substrate interface with a characteristic decay length of the order of *min*(*L*_*a*_, *L*_*c*_). The two limiting cases now read:

- In the limit $t\ll min({L}_{a},{L}_{c})$, the elastic energy is proportional to a volume $\sim {L}_{a}{L}_{c}t$ of the epitaxial layer and its expression is that obtained by Kaganer *et al*.^11^ in the limit of infinite lateral dimensions.
- In the limit $t\gg min({L}_{a},{L}_{c})$, the elastically strained layer is confined in a layer of thickness $\sim min({L}_{a},{L}_{c})$ above the interface. Hence, the elastic energy is proportional to a volume $\sim {L}_{a}{L}_{c}min({L}_{a},{L}_{c})$ and its relative importance with respect to bulk (volume) free energy vanishes as *min*(*L*_*a*_, *L*_*c*_)/*t*. One then expects to observe the same transition behavior as that observed in bulk MnAs, namely *α*-phase below *T*_*c*_ (in particular at ambient temperature *T*_0_ = *RT* < *T*_*c*_), and *β*-phase above.

Then, Eq. 6 is to be modified into:

7 $$\begin{array}{rcl}\sigma (T,\frac{t}{{L}_{min}}) & = & \frac{t}{{L}_{min}}\frac{1}{g(t/{L}_{min})}{\sigma }^{\infty }(T)\,{\rm{for}}\,{T}_{c}^{\ast }-{\rm{\Delta }}T\le T\le {T}_{c}^{\ast }\,{\rm{with}}\,\\ {\rm{\Delta }}T & = & \frac{{L}_{min}}{t}g(t/{L}_{min}){\rm{\Delta }}{T}^{\infty }\end{array}$$

where *L*_*min*_ = *min*(*L*_*a*_, *L*_*c*_) and *g*(*u*) is a dimensionless function presenting the same asymptotic limits as *f*(*u*). This allows us interpreting the observed effects of *L*_*c*_ on *σ* in Fig. 3(a) and the fact that larger *L*_*c*_ tends to yield smaller *σ* provided that *L*_*c*_ remains significantly smaller than *L*_*a*_

Back to the situation where *L*_*a*_ *L*_*c*_, Eq. 6 also permits to rationalize the effect of size reduction on the *α*/*β* phase coexistence temperature range (Fig. 5 top):

- As long as the lateral size along the high strain direction, *L*_*a*_, is large compared to the critical value ${L}_{a}^{c}=t/{u}_{c}\approx 1.8\,\mu m$, the behavior is that of a film with infinite lateral dimensions and the coexistence temperature range Δ*T* ^∞^ extends over ∼10 °C. This is *e*.*g*. the case when *L*_*a*_ = 12 *μm*.
- When *L*_*a*_ becomes small with respect to ${L}_{a}^{c}$, the coexistence temperature range is reduced and the behavior gets closer to that of bulk MnAs. This is *e*.*g*. the case when *L*_*a*_ = 0.75 *μm*. The coexistence temperature range is then predicted to be $\sim {L}_{a}{u}_{c}\Delta {T}^{\infty }/t$, which yields a value about ∼4 °C for *L*_*a*_ = 0.75 *μm*, in good agreement with the observations.

The above theoretical approach allows us interpreting the structure of *α* and *β* domains observed in Fig. 6. As the lateral size along the high strain direction is reduced, the behavior gets closer to that of bulk MnAs and pure *α* domains are observed at RT. As this lateral size is increased, *β* stripes start to develop and consequently the magnetic domains in the *α* phase tend to adopt the multidomain magnetic structure observed in the case of continuous MnAs thin films. It provides also a simple, qualitative interpretation of the effect of the shape (rectangles *vs* disks) on the domain structure (Fig. 6h): To optimize stress relaxation, the *α*/*β* wall boundaries are expected to cross the pattern edge perpendicularly. This will generate inclined walls in disks different from the parallel walls in rectangles (Fig. 5).

## Discussion

By combining local probe *μ*-XRD, LEEM and XMCD-PEEM measurements, we have shown that patterned MnAs/GaAs(001) samples can have very different microcrystalline, and consequently micromagnetic, behaviors; thin films results cannot be straightforwardly extrapolated to patterned microstructures.

We demonstrated that *α* and *β* phases coexist also for laterally confined geometries. The presence of the *α* and *β* MnAs phases was quantified and the influence of parameters like the microstructure shape, size, aspect ratio and orientation was studied as function of temperature, during the *α*/*β* phase transition.

Reducing the lateral dimensions of the MnAs microstructures along the large strain direction ($\mathrm{[11}\bar{2}\mathrm{0]}$) tends to stabilize the *α*-phase at RT. The temperature measurements allow to unambiguously evidence a strong variations of *T*_*c*_ and of the temperature range of the *α*/*β* phases coexistence as function of the lateral confinement. We highlight the important effect of the microstructure orientation parallel or perpendicular to the *α*/*β* stripes and the presence of a lateral critical size (${L}_{a}^{c}\simeq p\simeq 4.8t$). A theoretical (elastic based) model was developed, extending the model proposed by Kaganer *et al*.^11,12.^ The model predicts the ratio of *α*/*β* phases for microstructures with the lateral dimension varying in the direction parallel to the stripes and is in good agreement with the experimental results shown above (including at various temperatures).

This model is simple and purely two-dimensional, but it allows to give a very accurate description of finite size effects on the structural properties of MnAs microstructures. The present model could be further extended by taking into account the interfacial energies between *α* and *β* domains to explain size effects in both directions *i*.*e*. parallel and perpendicular to the *α*/*β* stripes. Indeed, work still has to be done to extend it in the perpendicular direction, where border effects and the presence of the *α*/*β* interface is expected to play a major role, as it has been evidenced in disk shaped microstructures. A possible direction for investigation should be the accurate knowledge of the *f*(*u*) function, for example *via* finite element simulations, for an accurate modeling of the strain relaxation inside the MnAs lithographed microstructures.

From the micromagnetic point of view, we found only an indirect effect of finite size reduction on the magnetic properties. The microstructures evidence the classified and well-known MnAs magnetic domains (Types I, II and III). As deduced from the *μ*-XRD measurements, below a critical size (${L}_{a}^{c}\mathrm{=1.5}\,\mu m$), the microstructures adopt predominantly the ferromagnetic *α*-phase. For the smallest microstructure size the *α*-phase develops a single domain state, similar to the one observed at low temperature in continuous thin films or in the bulk MnAs single crystal. Nevertheless, it is worth to notice that the microstructure magnetic domains are mostly influenced by edge effects rather than by finite size effects. Type I-III 3D flux-closure magnetic domains are formed at the edges of the microstructures, most probably to reduce the demagnetization energy. A possible extension of this work will be to perform 3D complex micromagnetic simulations to fully understand the edge effect on the stability of flux-closure 3D domains structures.

The presented structural, magnetic and theoretical modeling results are coherent and in perfect agreement. It is worth to notice that our structural results are in quite good agreement with those published by Tortarolo *et al*.^14^. However, we found a large disagreement with their micromagnetic results. A possible explanation of this disagreement could be the influence of the lithography processes on the magnetic properties of the MnAs microstructure (roughness, reactive and selective etching, mask influence..), especially as MnAs can be very chemically reactive^36,37^. In the present work and to overcome such limitations, we have always left a large area of the sample unpatterned (200 × 200 *μm* ^2^), but which also undergoes the full lithography processing. This large area serves as a reference for the continuous films and to check that the MnAs microstructures remain unaffected by the full patterning process. *μ*-XRD, *μ*-LEED (Low Energy Electron Diffraction), XAS (X-ray Absorption Spectroscopy), LEEM and XMCD-PEEM measurements have been simultaneously performed on these large areas under the same measurements conditions. The results of these measurements are in complete agreement with the published results for continuous MnAs thin films. Therefore we can confidently assess that our measurements are accurate and unaffected by the sample preparation methods. Finally, Tortarolo *et al*.^14^ have exclusively used MFM to characterize the magnetic domain structure. MFM is mainly sensitive to out-of-plane stray field and does not allow to straightforwardly determine the MnAs in-plane magnetic structure as it has been demonstrated in the case of the continuous MnAs film. Recently, Steren *et al*.^24^ have used XMCD-PEEM to study the micromagnetic changes in thin MnAs (30–50 nm) nano-ribbons, but they have not addressed the structural aspect nor the *α* − *β* phase coexistence. With respect to our conclusions, the magnetic changes observed in these nano-ribbons are more probably induced by edge effects than by size reduction effects.

To summarize, we have clearly demonstrated the strong influence of lateral confinement and size reduction on the structural and magnetic properties of MnAs microstructures. Our general finding is that the smaller are the microstructures, the more they resemble to the bulk infinite MnAs single crystal in terms of structural and magnetic properties. The microstructures adopt exclusively the *α*-phase at RT as in the case of the bulk MnAs, and the *α* − *β* phase coexistence temperature range is very reduced. From the magnetic point of view, the microstructures adopt predominantly ferromagnetic single mono-domains similar to the bulk MnAs. Finally, these experimental observations have been further confirmed by the elastic model, which demonstrates that when the size of the microstructures is much smaller than the film thickness (${L}_{a}\ll t$), the strain is limited to the interface and thus the microstructures behave like the bulk MnAs. All these results, obtained in the case of the prototypical MnAs system, confirm that size effects in microstructures can be very challenging to predict and have to be addressed very carefully.

## Methods

### Sample preparation and lithography methods

The samples were prepared at the AIST national institute (Tsukuba - Japan), in the so-called A-orientation following a well-established procedure using solid-source MBE (Molecular Beam Epitaxy)^26^. After thermal cleaning of the GaAs(100) substrate at 590 °C, a 40 nm GaAs buffer layer is grown at 570 °C. The MnAs layer is then grown at 210 °C with a growth rate of 5 nm per minute. In this orientation MnAs grows epitaxially adopting the following epitaxial relationship: MnAs$\mathrm{[1}\bar{1}\mathrm{00]//}$GaAs[001] and MnAs$\mathrm{[11}\bar{2}\mathrm{0]}$//GaAs$[\bar{1}\mathrm{10]}$. The films were post-annealed at 310 °C and As-capped to prevent any oxidation during the sample air transfer. The samples were decapped *in-situ* at 350 °C prior to the lithography process in order to remove the thick As protective layer. The samples were further capped with a 3 nm thick Ru layer to prevent any contamination of the surface during the lithography.

The MnAs microstructures have been patterned by electron beam lithography using a JEOL 6500F scanning electron microscope, using an Al mask and a subsequent Ar ion beam etching down to the GaAs substrate.

### Local probe X-Ray Diffraction

**Figure 10:**
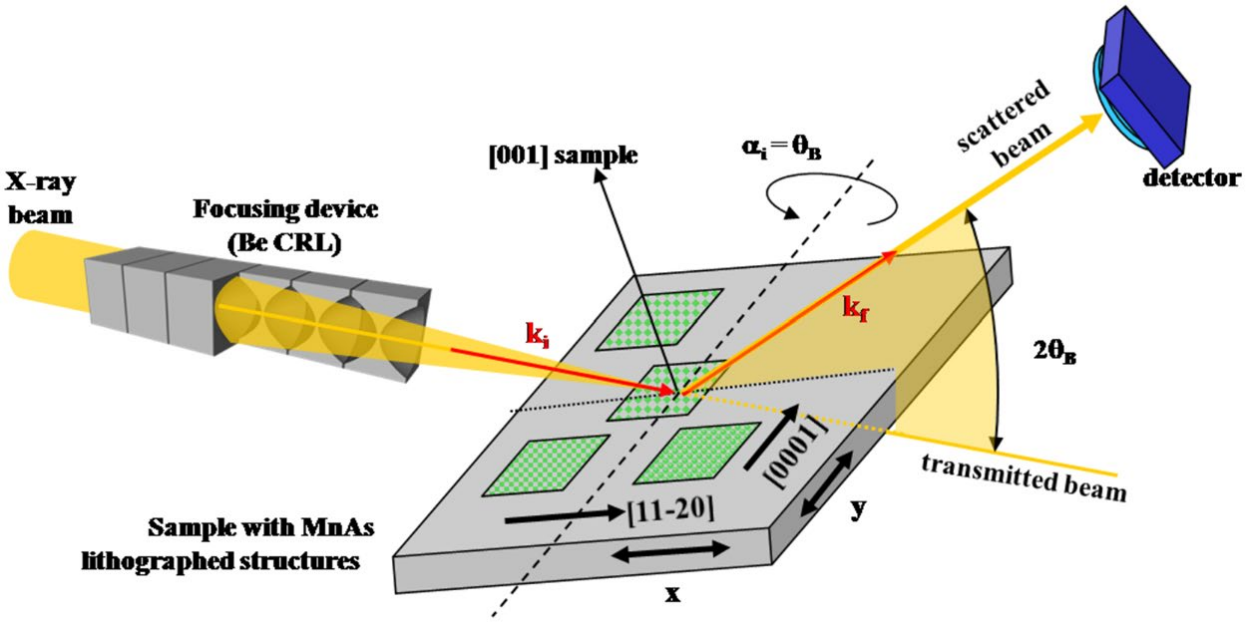
Schematic of the *μ*-XRD experimental setup using Be CRLs.

The XRD experiments detailed in the report were performed on the ID-01 beamline at the European Synchrotron Radiation Facility (ESRF), Grenoble, France and on the DiffAbs beamline at the Synchrotron SOLEIL, Gif-sur-Yvette, France (see Fig. 10). Various focusing devices were used: Be Compound refractive lenses (CRL)^38–41^ (at ESRF) or Kirkpatrick-Baez (KB) optics^42,43^ and Fresnel Zone Plate (FZP)^44–48^ (at the Synchrotron SOLEIL). The photon energy was in the 7.5 to 9.5 keV range, and the typical X-ray probe size was of about 1 × 3 *μm* ^2^ (vertical × horizontal) and 7 *μm* Full Width at Half Maximum of the intensity (FWHM). A X-ray hybrid pixel area detector (XPAD)^49–52^ was used to perform 3-dimensional mapping of the reciprocal space around the positions of the Bragg peaks characteristic for the MnAs layer (*α* and *β* phases).

The sample was mounted on a Peltier cooling/heating device, which allowed accessing the −15 °C to +60 °C temperature range. During the first experiments, the sample was kept under He flow to prevent possible oxidation and X-ray beam damage. It was found in later experiments that at lower photon fluxes, the sample can also be placed in air.

The experimental setup^53,54^ is depicted in Fig. 10. The angles of the diffractometer are set such to fulfill the diffraction (Bragg) condition for the MnAs film (*α* or *β* phases). Then, the sample lateral position is scanned, while recording with the detector the scattered intensity in each point. A raster image of the sample surface with crystallographic contrast is obtained^53–55^. The result is shown in Fig. 2-left and compared to the optical microscopy images of precisely the same area of the sample. The lateral resolution of the raster images in this approach is essentially given by the lateral size of the X-ray spot (its footprint on the sample), which was kept relatively large (few *μm*) on purpose, to integrate over several *α*/*β* stripes on the large objects, in order to obtain a result which is not related to the presence of possible local defects. Thus the reported structural data (quantity of *α* phase, strain, etc.) are characteristic for the probed object.

It is worth to notice, that the long crack lines running perpendicular to the [0001] direction are caused by the strain release. Along the [0001] direction, there is a large stress accumulation because there exist no stress reduction mechanism as for the $\mathrm{[11}\bar{2}\mathrm{0]}$ direction. For thick MnAs films (300 nm), the stress accumulation is so large that it induces the formation of periodic cracks extending over the whole MnAs film thickness down to the GaAs interface^26^.

### LEEM-PEEM microscopy

The high-resolution magnetic imaging experiments were performed on the French branch of the Nanospectroscopy beamline at the ELETTRA synchrotron facility (Trieste, Italy), using an Elmitec GmbH commercial LEEM/PEEM microscope (LEEM V). In the Low Energy Electron Microscopy (LEEM) mode^56^, elastically backscattered low energy electrons are used for imaging the surface. The lateral resolution of LEEM is better than 10 nm, and reveals the structural and morphologic features of the films. In the Photo Emission Electron Microscope (XPEEM) mode the microscope collects the secondary electrons emitted from the sample surface upon illumination by polarized and monochromatic X-rays, which in our case are incident on the sample at an angle of 16° from the surface and form a 10 × 10 *μm* ^2^ beam spot. The spatial resolution of the microscope in the XPEEM mode is limited by the chromatic and spherical aberrations to 25 nm. The probing depth is very small, in general below 10 nm, due to the small inelastic mean free path of the secondary photoelectrons.

The micromagnetic spin structure of the *α*-MnAs surface was determined taking advantage of the large XMCD effect associated with the Mn *L*_3_ edge using circular-polarization light^57^. In the XMCD-PEEM method, the electron yield difference between opposite helicities of the photon beam is proportional to the dot product of the magnetization and the direction of the photon beam, which enables the mapping of the essentially in-plane component of surface magnetization. The samples were mounted with the MnAs magnetic in-plane easy axis aligned in the plane of incidence of the photon beam in order to optimize the magnetic contrast within the *α*-stripes.

Prior to the LEEM-PEEM experiment, the sample has been Argon ion etched in order to remove the residual polymer mask from the lithography and the Ru capping layer. The sample has been further annealed well below the original deposition temperature (200 °C) in order to recover the MnAs surface crystalline structure as it has been evidenced using LEEM (see Fig. 6a) and *μ*-LEED measurements (not shown).

### Determination of the dimensionless function *f(u)* used in Eqs 4 to 6

**Figure 11:**
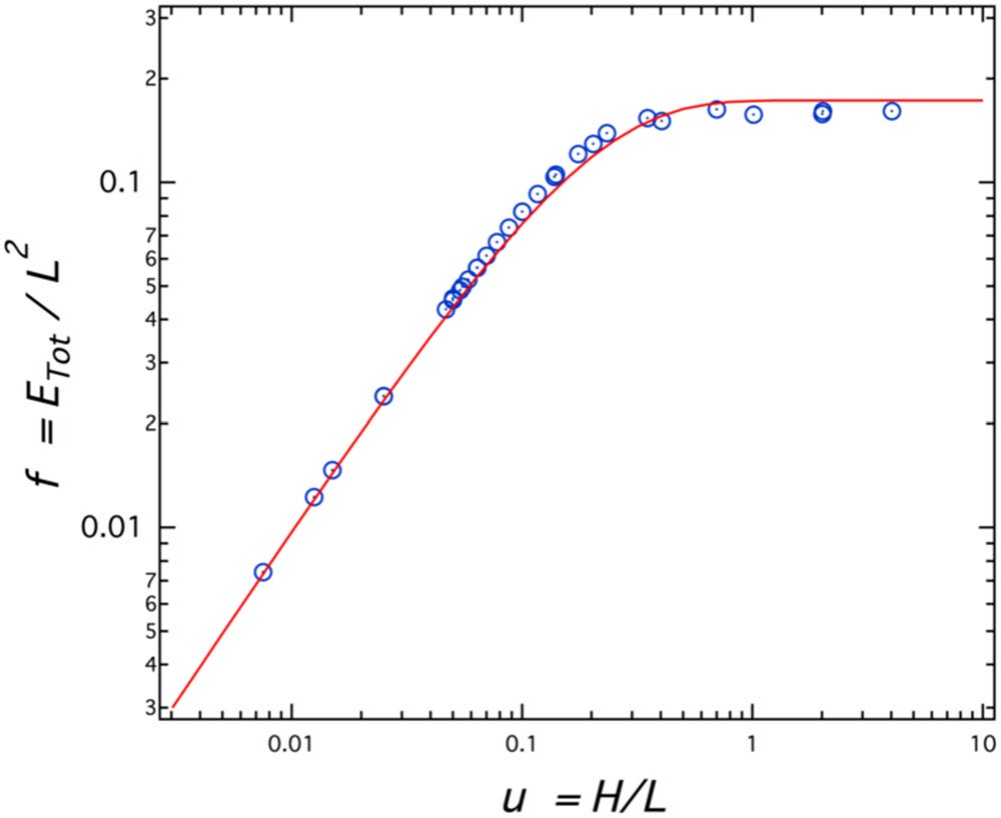
Variation of the dimensionless function *f* = *E*_*tot*_/*L* ^2^ as a function of the aspect ratio H/L in a triangular spring network of size L × H loaded by imposing a constant strain at the bottom edge. Here, *E*_*tot*_ refers to the total elastic energy stored in all connected springs. Open circles are the results of the simulation. The red plain curve corresponds to *f*(*u*) = *u*_*c*_(1 − *exp*(−*u*/*u*_*c*_)), where the fitted parameter is found to be *u*_*c*_ = 0.166 ± 0.005 within a 99% confident level.

The function *f*(*u*) characterizing the dependence of the elastic energy embedded in the epitaxial layer on its aspect ratio has been computed by means of central force networks: Nodes connected by springs of unit stiffness are placed on a two-dimensional triangular lattice of horizontal and vertical dimensions L and H, respectively. Such a network, indeed, obeys Hookean linear elasticity with a Young modulus $Y=2/\sqrt{3}$ and a Poisson ratio *ν* = 1/3. A horizontal strain of unit value is then applied to the node at the bottom and the positions equilibrating all the forces are determined at all the nodes. The total elastic energy *E*_*tot*_ is finally computed as the sum of the energy stored in all the springs. The plot *E*_*tot*_/*L* ^2^ as a function of H/L provides the function *f*(*u*) (Fig. 11).
